# Supplementary material for: DNA methylation analyses identify an intronic ZDHHC6 locus associated with time to recurrent stroke in the Vitamin Intervention for Stroke Prevention (VISP) clinical trial
Source: PLoS One. 2021 Jul 12;16(7):e0254562. doi: 10.1371/journal.pone.0254562 (PMC8274879; doi:10.1371/journal.pone.0254562)
Supplement: S1 Table — (DOCX) [file pone.0254562.s001.docx]

**S1 Table. List of blood biomarkers and clinical traits used in WGCNA.**

| **Biomarkers and Trait Names** | **Unit of Measure/Response** |
| --- | --- |
| VISP recurrent stroke endpoint | Yes/No |
| Time to VISP recurrent stroke | Days |
| Composite vascular endpoint | Yes/No |
| Time to composite vascular endpoint | Days |
| Previous number of strokes | Number of events |
| Recurrent stroke ever status | Yes/No |
| Modified Rankin stroke scale | Range from 0-3 |
| Myocardial infarction status | Yes/No |
| Diabetes mellitus status | Yes/No based on self-report |
| Hypertension status | Yes/No based on self-report |
| Systolic blood pressure | mmHg |
| Diastolic blood pressure | mmHg |
| B6 | nmol/L |
| B12 | pmol/L |
| Folate | ng/mL |
| Total plasma homocysteine level | µmol/L |
| Creatinine | mg/dL |
| Total cholesterol | mg/dL |
| High-density lipoprotein | mg/dL |
| Triglycerides | mg/dL |
| C-reactive protein | mg/L |
| Prothrombin fragments 1+2 | nmol/L |
| Thrombin-antithrombin complex | µg/L |
| Thrombomodulin | ng/mL |
| Tissue plasminogen activator | ng/mL |
| von Willebrand Factor | IU/L |
