# Supplementary material for: DNA methylation analyses identify an intronic ZDHHC6 locus associated with time to recurrent stroke in the Vitamin Intervention for Stroke Prevention (VISP) clinical trial
Source: PLoS One. 2021 Jul 12;16(7):e0254562. doi: 10.1371/journal.pone.0254562 (PMC8274879; doi:10.1371/journal.pone.0254562)
Supplement: S2 Table — (DOCX) [file pone.0254562.s002.docx]

**S2 Table. Gene ontology results from significant methylation loci.**

| **Category** | **GO term** | **Description** | **P** | **FDR** | **^a^Enrichment** | **N** | **B** | **n** | **b** |
| --- | --- | --- | --- | --- | --- | --- | --- | --- | --- |
| BP | GO:0060134 | prepulse inhibition | 8.22E-06 | 0.13 | 436.93 | 18351 | 12 | 7 | 2 |
| BP | GO:0048854 | brain morphogenesis | 2.87E-05 | 0.22 | 238.32 | 18351 | 22 | 7 | 2 |
| BP | GO:0033032 | regulation of myeloid cell apoptotic process | 3.14E-05 | 0.16 | 227.96 | 18351 | 23 | 7 | 2 |
| BP | GO:0014065 | phosphatidylinositol 3-kinase signaling | 8.25E-05 | 0.32 | 141.71 | 18351 | 37 | 7 | 2 |
| BP | GO:0051898 | negative regulation of protein kinase B signaling | 2.11E-04 | 0.65 | 88.87 | 18351 | 59 | 7 | 2 |
| BP | GO:2000106 | regulation of leukocyte apoptotic process | 3.51E-04 | 0.90 | 68.99 | 18351 | 76 | 7 | 2 |
| BP | GO:0048015 | phosphatidylinositol-mediated signaling | 3.60E-04 | 0.79 | 68.09 | 18351 | 77 | 7 | 2 |
| BP | GO:0022603 | regulation of anatomical structure morphogenesis | 3.77E-04 | 0.73 | 9.6 | 18351 | 1092 | 7 | 4 |
| BP | GO:2000808 | negative regulation of synaptic vesicle clustering | 3.81E-04 | 0.65 | 2,621.57 | 18351 | 1 | 7 | 1 |
| BP | GO:0048017 | inositol lipid-mediated signaling | 3.98E-04 | 0.61 | 64.73 | 18351 | 81 | 7 | 2 |
| BP | GO:0006661 | phosphatidylinositol biosynthetic process | 5.83E-04 | 0.82 | 53.5 | 18351 | 98 | 7 | 2 |
| BP | GO:0051128 | regulation of cellular component organization | 6.94E-04 | 0.89 | 5.36 | 18351 | 2444 | 7 | 5 |
| BP | GO:0007409 | axonogenesis | 6.94E-04 | 0.82 | 49 | 18351 | 107 | 7 | 2 |
| BP | GO:0051548 | negative regulation of keratinocyte migration | 7.63E-04 | 0.84 | 1,310.79 | 18351 | 2 | 7 | 1 |
| BP | GO:0060024 | rhythmic synaptic transmission | 7.63E-04 | 0.78 | 1,310.79 | 18351 | 2 | 7 | 1 |
| BP | GO:0050793 | regulation of developmental process | 8.74E-04 | 0.84 | 5.11 | 18351 | 2566 | 7 | 5 |
| BP | GO:0001952 | regulation of cell-matrix adhesion | 8.86E-04 | 0.80 | 43.33 | 18351 | 121 | 7 | 2 |
| BP | GO:0014066 | regulation of phosphatidylinositol 3-kinase signaling | 9.75E-04 | 0.84 | 41.28 | 18351 | 127 | 7 | 2 |
| MF | GO:0051800 | phosphatidylinositol-3,4-bisphosphate 3-phosphatase activity | 3.81E-04 | 1.00 | 2,621.57 | 18351 | 1 | 7 | 1 |
| MF | GO:0051717 | inositol-1,3,4,5-tetrakisphosphate 3-phosphatase activity | 7.63E-04 | 1.00 | 1,310.79 | 18351 | 2 | 7 | 1 |
| ^a^ Enrichment= (b/n)/(B/N)  **Abbreviations**: GO- gene ontology; FDR- false discovery rate q-value; N- total number of genes; B- total number of genes associated with GO term; n- number of genes in the target set; b- number of genes in the intersection; BP- biological process; MF- molecular function | | | | | | | | | |
