# Supplementary material for: DNA methylation analyses identify an intronic ZDHHC6 locus associated with time to recurrent stroke in the Vitamin Intervention for Stroke Prevention (VISP) clinical trial
Source: PLoS One. 2021 Jul 12;16(7):e0254562. doi: 10.1371/journal.pone.0254562 (PMC8274879; doi:10.1371/journal.pone.0254562)
Supplement: Attachment — Submitted filename: Response to Reviewers.docx [file pone.0254562.s003.docx]

The authors express their appreciation and gratitude to the editor and reviewers for their thoughtful critiques. Below, please find our itemized responses which correspond to **bold underlined text** and corresponding tracked changes throughout the manuscript.

**From Editor:**

When submitting your revision, we need you to address these additional requirements.

1. Please ensure that your manuscript meets PLOS ONE's style requirements, including those for file naming. The PLOS ONE style templates can be found at

<https://journals.plos.org/plosone/s/file?id=wjVg/PLOSOne_formatting_sample_main_body.pdf> and

<https://journals.plos.org/plosone/s/file?id=ba62/PLOSOne_formatting_sample_title_authors_affiliations.pdf>

**Response:** We have formatted our documents to meet PLOS ONE’s style requirements.

2. Please review your reference list to ensure that it is complete and correct. If you have cited papers that have been retracted, please include the rationale for doing so in the manuscript text, or remove these references and replace them with relevant current references. Any changes to the reference list should be mentioned in the rebuttal letter that accompanies your revised manuscript. If you need to cite a retracted article, indicate the article’s retracted status in the References list and also include a citation and full reference for the retraction notice.

**Response:** References have been reviewed and are deemed complete and correct. See itemized comments below.

3. We note that you are reporting an analysis of a microarray, next-generation sequencing, or deep sequencing data set. PLOS requires that authors comply with field-specific standards for preparation, recording, and deposition of data in repositories appropriate to their field. Please upload these data to a stable, public repository (such as ArrayExpress, Gene Expression Omnibus (GEO), DNA Data Bank of Japan (DDBJ), NCBI GenBank, NCBI Sequence Read Archive, or EMBL Nucleotide Sequence Database (ENA)). In your revised cover letter, please provide the relevant accession numbers that may be used to access these data. For a full list of recommended repositories, see [http://journals.plos.org/plosone/s/data-availability#loc-omics](about:blank#loc-omics) or [http://journals.plos.org/plosone/s/data-availability#loc-sequencing](about:blank#loc-sequencing).

**Response:** In the original text (Methods section) we state: Individual level genetics and epigenetics data is considered sensitive controlled data and cannot be shared publicly, as specified by the IRBs of Wake Forest School of Medicine, the University of North Carolina at Chapel Hill School of Medicine, and the University of Virginia School of Medicine, along with the NIH Data Access Committee. Controlled-access data can only be obtained if a user has been authorized by the appropriate Data Access Committee. The individual level Genomics and Randomized Trials Network (GARNET) VISP data are available in the database of Genotypes and Phenotypes (dbGaP) (Accession: phs000343.v3.p1). The authors will also share the data on request.

Additionally, we added the following line in the Replication Cohort section within the Methods regarding data accessibility of the replication data: “The epigenetics and genetics summary data used for replication will be shared for research studies on approval of the principal investigators of the GRECOS (Genotyping Recurrence Risk of Stroke) cohort.”

**Comments to the Author:**

1. Is the manuscript technically sound, and do the data support the conclusions?

The manuscript must describe a technically sound piece of scientific research with data that supports the conclusions. Experiments must have been conducted rigorously, with appropriate controls, replication, and sample sizes. The conclusions must be drawn appropriately based on the data presented.

Reviewer #1: Yes

Reviewer #2: Yes

Reviewer #3: Yes

2. Has the statistical analysis been performed appropriately and rigorously?

Reviewer #1: I Don't Know

Reviewer #2: Yes

Reviewer #3: Yes

3. Have the authors made all data underlying the findings in their manuscript fully available?

The [PLOS Data policy](https://nam02.safelinks.protection.outlook.com/?url=http%3A%2F%2Fwww.plosone.org%2Fstatic%2Fpolicies.action%23sharing&data=04%7C01%7CKEENEK%40ECU.EDU%7C2254a1904d1a47ec364308d912b9a682%7C17143cbb385c4c45a36ac65b72e3eae8%7C0%7C1%7C637561411166906425%7CUnknown%7CTWFpbGZsb3d8eyJWIjoiMC4wLjAwMDAiLCJQIjoiV2luMzIiLCJBTiI6Ik1haWwiLCJXVCI6Mn0%3D%7C1000&sdata=cLyWC3ncDtut15Xr%2BNqEJe7aMq5VONWcdOjyENQin8g%3D&reserved=0) requires authors to make all data underlying the findings described in their manuscript fully available without restriction, with rare exception (please refer to the Data Availability Statement in the manuscript PDF file). The data should be provided as part of the manuscript or its supporting information, or deposited to a public repository. For example, in addition to summary statistics, the data points behind means, medians and variance measures should be available. If there are restrictions on publicly sharing data—e.g. participant privacy or use of data from a third party—those must be specified.

Reviewer #1: No

Reviewer #2: Yes

Reviewer #3: Yes

4. Is the manuscript presented in an intelligible fashion and written in standard English?

PLOS ONE does not copyedit accepted manuscripts, so the language in submitted articles must be clear, correct, and unambiguous. Any typographical or grammatical errors should be corrected at revision, so please note any specific errors here.

Reviewer #1: Yes

Reviewer #2: Yes

Reviewer #3: Yes

5. Review Comments to the Author

Please use the space provided to explain your answers to the questions above. You may also include additional comments for the author, including concerns about dual publication, research ethics, or publication ethics. (Please upload your review as an attachment if it exceeds 20,000 characters)

Reviewer #1: Dear author(s)
first, I would like to congratulate you for the tremendous work that has been established in this paper, and I am terribly sorry for the loss of one of the authors.
there are very minor points which I would like to suggest:

**Response:** Thank you for your acknowledgement regarding the tremendous work and your condolences.

1. RESULTS; line 211. it is written scompared, please correct.

**Response:** We have made the appropriate edit.

2. REFERENCE: please edit the fonts style to be similar with the rest of text.

**Response:** We have made the appropriate edits to the references.

3. FIGURES: please change the resolution of the figures as they are blurry and not clear. please cite the figures in text as (Fig 1) ...etc.

**Response:** We improved the quality of the figures and uploaded the figures to the PLOS Preflight Analysis and Conversion Engine (PACE) digital diagnostic tool to ensure they meet the PLOS requirements. Additionally, we addressed the figure citation in the text accordingly.

4. STYLE: please justify the text within and remove any unnecessary spaces

**Response:** We have formatted the text accordingly by justifying the text and removing unnecessary spaces.

5. TABLES: kindly edit the tables captions font size and style. (refer to the guidelines)

**Response:** We have edited the table and figure captions, titles, font, and style accordingly.

6. ETHICS: if you have any ethics code, please state it in the ETHICS section.

**Response:** No additional ethics codes, beyond our IRB statements have been included.

7. Supporting information captions should be added after the REFERENCES section. Add Supporting information heading.

**Response:** We have added the Supporting information heading and information after the REFERENCES section.

Reviewer #2: Summary:
The article describes a novel approach to elucidate the impact of epigenetic markers in disease, more specifically in recurrent stroke. The arguments are well structured, the paper is clear and the conclusions are important to the field. However, the cohort is too small to reach relevant conclusions and the heterogenicity of blood samples may need a bigger cohort to achieve consensus in the field.

Minor points:

1.- The quality of the figures is too low. I would recommend to improve it prior publication.

**Response:** We improved the quality of the figures and uploaded the figures to the PLOS Preflight Analysis and Conversion Engine (PACE) digital diagnostic tool to ensure they meet the PLOS requirements.

2.- Descriptions in figures and tables are vague.

**Response:** We have edited the table and figure captions, titles, font and style accordingly.

3.- The criteria for the loci chosen for analysis is missing.

**Response:** We have edited the text as follows (Methylation Data Generation section):

“Illumina Infinium Human Methylation450k BeadChip microarrays were used to interrogate CpG sites across the genome and the resulting intensity files were analyzed using GenomeStudio. Beta scores were generated as the ratio of methylated intensities divided by the sum of the methylation and unmethylated intensities (i.e. the proportion of total signal from the methylation specific probe). Probes were removed if they failed to hybridize (detection p>5%) or if they were located on sex chromosomes. The filtered beta scores underwent stratified quantile normalization with the *minfi* package in R [11-13]. A total of 470,871 autosomal CpGs were used in subsequent analyses.”

4.- In conclusions, an hypothesis to explain the differences between cohorts is missing.

**Response:** We have edited the text as follows (Discussion section):

“Furthermore, previous studies have shown that global methylation patterns differ across ethnicities, as well as within ethnic subgroups. In a study of 573 individuals from diverse Latio ethnic sub-groups, genetic ancestry explained approximately 75% of the variation in methylation between the sub-groups [52]. Therefore, the use of cohorts from different race/ethnicities should allow for the identification of methylation sites with global implications (affecting most ethnicities similarly) but will limit our ability to identify race/ethnicity-specific associations.

Major points:

1.- The paper describes seven significant loci identified by methylation analysis. However, a deep analysis for those specific regions is missing. I recommend the inclusion of extra figures/ supplementary data highlighting the epigenetic characteristics of those regions, including a map of the genes nearby, with annotations of , for example, SUZ12 and histone modification marks, to inquire into the relevance of the regions reported and the origin of the methylation differences.

Available datasets could help to generate extra figures to show histone modification marks, methylation profiles and GWAS enrichment in the regions reported. Also, to use a primary cell line of relevance to do a ChIP-seq of interesting marks could help to show the “normal” methylation profile and epigenetic characteristics that we would expect in healthy population without the bias of the blood heterogeneity and then we would be able to compare it to the reported cohort.

**Response:** We have added additional *in silico* data to help further elucidate any biological implications of our findings. The ENCODE annotation and gene position for the most significant site, cg03584380 is presented in Fig 2. We performed functional annotation and mapping of the 57 unique genes with suggestive (p<1.00E-06) CpG loci from the Cox PH results on AFR VISP recurrent stroke and composite vascular endpoint analyses. Tissue analysis on 53 specific types from the GTEx project, revealed statistically significant differential down-regulated expression in the pancreas (p_adj_=3.05e-08), putamen basal ganglia (p_adj_=2.42e-06), left ventricle of the heart (p_adj_=1.54e-05), liver (p_adj_=8.75e-05), amygdala (p_adj_=1.50e-04), caudate basal ganglia (p_adj_=2.30e-04), hippocampus (p_adj_=3.52e-04), nucleus accumbens basal ganglia (p_adj_=9.30e-04), anterior cingulate cortex BA24 (p_adj_=5.93e-04), substantia nigra (p_adj_=6.02e-03), skeletal muscle (p_adj_=2.72e-02), whole blood (p_adj_=2.91e-02), hypothalamus (p_adj_=2.93e-02), and the cortex (p_adj_=3.89e-02) (Fig 5, Table 4).

Reviewer #3: The MS is sound and I can not visualize any new experiment which could be necessary to strenght the conclusions.The paper is solid and the experiments were well thought.Even if it s a descriptive paper it has enough merits to be published

6. PLOS authors have the option to publish the peer review history of their article ([what does this mean?](https://nam02.safelinks.protection.outlook.com/?url=https%3A%2F%2Fjournals.plos.org%2Fplosone%2Fs%2Feditorial-and-peer-review-process%23loc-peer-review-history&data=04%7C01%7CKEENEK%40ECU.EDU%7C2254a1904d1a47ec364308d912b9a682%7C17143cbb385c4c45a36ac65b72e3eae8%7C0%7C1%7C637561411166906425%7CUnknown%7CTWFpbGZsb3d8eyJWIjoiMC4wLjAwMDAiLCJQIjoiV2luMzIiLCJBTiI6Ik1haWwiLCJXVCI6Mn0%3D%7C1000&sdata=uD1QE8yumMxwqPaE3LOuNZP6oYOlP8MHHLun7aLN3Ls%3D&reserved=0)). If published, this will include your full peer review and any attached files.


If you choose “no”, your identity will remain anonymous but your review may still be made public.


**Do you want your identity to be public for this peer review?** For information about this choice, including consent withdrawal, please see our [Privacy Policy](https://nam02.safelinks.protection.outlook.com/?url=https%3A%2F%2Fwww.plos.org%2Fprivacy-policy&data=04%7C01%7CKEENEK%40ECU.EDU%7C2254a1904d1a47ec364308d912b9a682%7C17143cbb385c4c45a36ac65b72e3eae8%7C0%7C1%7C637561411166916422%7CUnknown%7CTWFpbGZsb3d8eyJWIjoiMC4wLjAwMDAiLCJQIjoiV2luMzIiLCJBTiI6Ik1haWwiLCJXVCI6Mn0%3D%7C1000&sdata=5E6LwI3Y09lqxsQvSXPlPcD4LtLO5PMqFW7kYFpqj3Y%3D&reserved=0).

Reviewer #1: **Yes:** Dr Noora R. Al-Snan

Reviewer #2: No

Reviewer #3: No
